# Harnessing generative AI for predicting and optimizing antimicrobial peptides against drug-resistant infections

**DOI:** 10.1038/s44259-026-00218-3

**Published:** 2026-05-22

**Authors:** Sandra Clemens, Hannah Franziska Löchel, Nico Häußer, Felix Wannemacher, Wilhelm Bertrams, Bernd Schmeck, Dominik Heider

**Affiliations:** 1 Institute of Medical Informatics, University of Münster, Münster, Germany; 2 Department of Mathematics and Computer Science, Philipps-University Marburg, Marburg, Germany; 3 Institute for Computer Science, Heinrich-Heine-University Düsseldorf, Düsseldorf, Germany; 4 Institute for Lung Research, Universities of Giessen and Marburg Lung Center, German Center for Lung Research (DZL), Philipps-University Marburg, Marburg, Germany; 5 Center for Synthetic Microbiology (SYNMIKRO), Philipps-University Marburg, Marburg, Germany; 6 Institute for Lung Health (ILH), Giessen, Germany; 7 Member of the German Center for Infectious Disease Research (DZIF), Marburg, Germany

**Keywords:** Chemistry, Computational biology and bioinformatics, Drug discovery

## Abstract

Antimicrobial resistance (AMR) poses a major global health threat that demands the discovery of new antimicrobial agents. Antimicrobial peptides (AMPs) offer a promising therapeutic alternative due to their broad-spectrum activity and reduced likelihood of resistance development. In the current study, we developed COMPASS, a comprehensive database aggregating 75,381 unique AMP sequences from nine public repositories, and created AmpGPT2, a transformer-based generative model specifically fine-tuned for AMP sequence generation. Unlike directed approaches, which optimize antimicrobial sequences or certain properties, our foundational model learns general AMP sequence patterns through an undirected training strategy. AmpGPT2 generated peptide sequences, of which 95.41% were predicted to be AMPs by AMP Scanner, representing a substantial improvement over existing models. The generated peptides exhibit physicochemical properties consistent with natural AMPs, including appropriate length distributions and molecular characteristics. Experimental validation demonstrated that one of five tested peptides, which shares structural features with dermaseptin-family AMPs, exhibited significant concentration-dependent antimicrobial activity against *Klebsiella pneumoniae* and *Pseudomonas aeruginosa*, supporting the model’s potential for functional AMP discovery. Highlighting the persistent challenge of translating computational predictions into biological function, this work establishes a foundational framework for AMP discovery that can serve as a basis for subsequent directed optimization strategies, potentially accelerating the development of novel antimicrobial therapeutics.

## Introduction

According to the WHO, antimicrobial resistance (AMR) represents a global threat to health, food security, and societal development, driven by the widespread overuse of antibiotics. This has facilitated the emergence of multidrug-resistant pathogens, which compromise the effectiveness of current treatments for infectious diseases^1^. Without countermeasures, up to 10 million people could die from AMR each year by 2050. The growing prevalence of resistance has created an urgent need for new therapeutic strategies. However, the development of new antibiotics is a challenge both from a scientific and economic perspective, due to high costs, regulatory hurdles, and low profitability. Antimicrobial peptides (AMPs) have emerged as a promising alternative due to their ability to target pathogens through multiple mechanisms, thus reducing the likelihood of resistance development^2^. AMPs are small, naturally occurring peptides that typically consist of fewer than 100 amino acids^3^. They play a critical role in innate immunity across diverse organisms, acting as a first line of defense against a broad spectrum of pathogens, including bacteria, viruses, fungi, and even cancer cells^2,4^. By disrupting microbial membranes^5^, modulating immune responses^6^, and acting synergistically with conventional antibiotics^7^, AMPs provide a multifaceted approach that positions them as a strong candidate in the fight against drug-resistant pathogens^8^. Similarly, natural antimicrobial compounds, such as chlorotonils, have been shown to simultaneously target bacterial membranes and protein synthesis pathways, representing multiple target mechanisms that hinder resistance development^9^.

Despite their promise, the discovery of new AMPs remains a challenging and resource-intensive task^10^. Traditional approaches, which are based on biological assays and experimental screening, are time-consuming and limited in scope^11,12^. The vast diversity of AMPs in nature, coupled with the subtle sequence and structural variations that influence their activity, further increases the complexity of the discovery of AMP^13,14^.

Recent advances in machine learning (ML) and natural language processing (NLP) have opened new possibilities for the discovery of antibiotics and AMPs. For example, Swanson et al.^15^ introduced SyntheMol, a generative AI model designed to produce easily synthesized and structurally novel antibiotics. To achieve this, they employed Monte Carlo Tree Search to explore the combinatorial chemical space and trained the model on antibiotic activity using a molecular property prediction approach. Similarly, Li et al.^16^ developed deepAMP, a generative deep learning model for peptides. The model leverages a BERT-like architecture and was trained on peptides with established membrane-disrupting activity, that is, one of the modes of action of AMPs. Their framework takes peptides with a known mechanism of action as input and generates optimized variants with enhanced antimicrobial activity.

These approaches enable the prediction and generation of novel peptides using computational methods^17,18^. Within this domain, transformer-based language models, originally developed for NLP tasks, have demonstrated significant potential in bioinformatics^19^, where protein sequences can be analyzed as a “biological language"^18^. ProtGPT2^18^, a generative transformer model based on GPT-2^17^, represents a major advancement in this field, producing de novo protein sequences by learning from extensive protein databases. ProtGPT2 generates sequences by autoregressively predicting each amino acid from its preceding context, thereby capturing intricate relationships within the sequence that mirror the principles of natural protein folding and function^18^.

While ProtGPT2 demonstrates the potential of transformer-based models for protein sequence generation, it is not tailored to AMPs. Thus, in the current study, our aim was to develop both COMPASS (Collection of Antimicrobial Peptide Databases), a curated database of AMP sequences, and AmpGPT2, a transformer-based model fine-tuned for AMP discovery. COMPASS is a publicly available resource that provides a curated dataset of AMP sequences aggregated from multiple sources into a unified, accessible platform designed for computational AMP discovery. The COMPASS interface is implemented in React to support machine learning applications, offering a reliable foundation for AMP research and enabling researchers to leverage a large, well-structured set of AMP sequences for training and analysis. This platform serves not only as a critical resource for AMP-related studies but also as a tool for efficiently applying machine learning methods to generate and evaluate novel peptides. Building on data from COMPASS, we developed AmpGPT2, a model specifically fine-tuned for AMP discovery across different modes of action. By fine-tuning ProtGPT2 on COMPASS, AmpGPT2 generates sequences tailored for AMP discovery, thereby enhancing its ability to produce novel peptides with potential antimicrobial properties. As a proof-of-concept, we evaluated the model’s capacity to generate plausible AMPs and performed experimental validation by testing a small set of AmpGPT2-generated candidates against clinically relevant bacterial strains, including *Klebsiella pneumoniae*, *Pseudomonas aeruginosa*, and *Streptococcus pneumoniae*. These species are clinically important pathogens associated with pneumonia and other severe infections. Notably, *Klebsiella pneumoniae* and *Pseudomonas aeruginosa* are Gram-negative bacteria classified among the WHO ESKAPE pathogens due to rising antimicrobial resistance^20–22^. Therefore, identifying alternative therapeutic strategies to combat infections caused by these organisms is of critical importance.

Together, COMPASS and AmpGPT2 provide a comprehensive approach to AMP discovery, combining curated datasets with state-of-the-art language models to address traditional limitations in AMP research and facilitate the development of new antibiotics.

## Results

### COMPASS

**Fig. 1:**
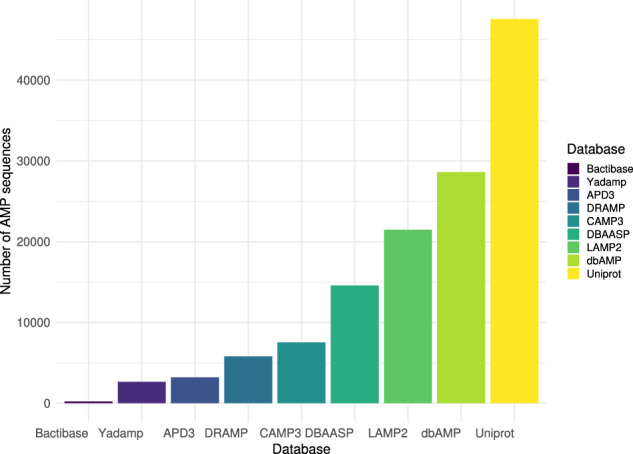
Number of AMP sequences collected from each database and included in COMPASS. The databases shown (Bactibase, YADAMP, APD3, DRAMP, CAMP3, DBAASP, LAMP2, dbAMP, and UniProt) are arranged in ascending order, and the height of each bar represents the total number of unique AMP sequences.

COMPASS contains 75,381 unique sequences, each assigned a unique identifier beginning with “COM” followed by a five-digit number. Only three sequences are present in all nine databases: Divergicin m35 (COM00066), Mundticin (COM00069), and Aureocin a53 (COM00133). Figure 1 shows the number of AMPs collected from each database and integrated into COMPASS. No sequences were found exclusively in Bactibase, APD3, or LAMP2, respectively. The number of sequences found exclusively in other databases is: CAMP3 (27), dbAMP (1868), DRAMP (65), UniProt (44,840), YADAMP (14), and DBAASP (1704).

The supplementary material additionally includes a Venn diagram (Fig. S1), a heatmap (Fig. S2), and an UpSet plot (Fig. S3), illustrating the intersections of the sets of AMPs within the databases. The complete database is available for download at https://compass.imi.uni-muenster.de/data.json, and a web interface can be accessed at https://compass.imi.uni-muenster.de.

### AmpGPT2

The AmpGPT2 model is available at https://imigitlab.uni-muenster.de/heiderlab/ampgpt2. Table 1 shows the percentage of sequences generated by ProtGPT2 and our fine-tuned AmpGPT2 classified as AMPs by AMP Scanner and CAMP. Out of 10,000 sequences generated by ProtGPT2, 55.3% are predicted to be AMPs by AMP Scanner and 33.89%-55.3% by CAMP, respectively. In contrast, AmpGPT2 yielded 95.41% and 70%–80% predicted AMPs, respectively. For comparison, our training and validation datasets had between 70% and 80% of AMP-predicted peptides. The AMP Scanner training data overlaps with our training data by 6%, while CAMP has a potential overlap of 17%. ProtGPT2-generated sequences showed the highest token- and sequence-level entropy (6.06 nats and 6.16 nats, respectively). AmpGPT2-generated sequences exhibited lower entropy (2.83 nats and 3.02 nats). Training and validation sequences for AmpGPT2 had similar entropy values (3.48 nats to 3.57 nats). Negative log-likelihood values followed the same pattern across sequence sets.

**Table 1:** AMP prediction between AmpGPT2 and ProtGPT2 generated sequences, as well as our training and validation data (percentages only)

Data	AMP Scanner	CAMP SVM	CAMP RF	CAMP NN	CAMP DA
AmpGPT2	95.41%	79.70%	76.01%	70.70%	71.15%
ProtGPT2	55.30%	42.93%	51.25%	33.89%	38.72%
Train	82.67%	74.60%	72.45%	69.90%	69.73%
Validation	82.92%	73.75%	72.99%	69.93%	70.39%

**Fig. 2:**
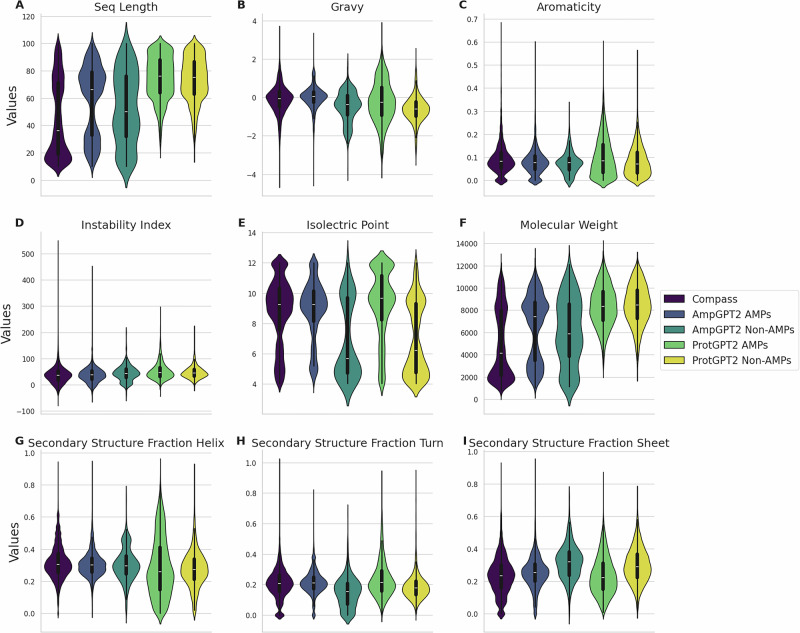
Distribution of peptide length and calculated physicochemical properties across natural and generated AMP datasets. Violin plots compare COMPASS antimicrobial peptides, AmpGPT2-generated peptides classified as AMPs or non-AMPs by AMP Scanner, and ProtGPT2-generated peptides classified as AMPs or non-AMPs by AMP Scanner. Each violin shows the distribution of values for one dataset. **A** peptide length distribution. **B** GRAVY score, indicating average hydrophobicity. **C** aromaticity, representing the fraction of aromatic residues. **D** instability index, estimating peptide stability. **E** isoelectric point, indicating the predicted pH at which the peptide has no net charge. **F** molecular weight. **G** fraction of residues with helix-forming propensity. **H** fraction of residues with turn-forming propensity. **I** fraction of residues with sheet-forming propensity.

Figure 2A presents the length distributions of AMP sequences from the COMPASS dataset, besides sequences generated by AmpGPT2 and ProtGPT2, respectively. For both generative models, the distributions are separated into sequences identified as potential AMPs and non-AMPs. ProtGPT2 produces generally longer sequences than AmpGPT2, whereas AmpGPT2’s length distribution more closely resembles that of the COMPASS database, with a higher proportion of sequences between 10 and 40 amino acids and a secondary peak between 60 and 80 amino acids. Figure 2B-I summarizes common physicochemical properties, including GRAVY (hydropathy), aromaticity, instability index, isoelectric point, molecular weight, and secondary structure fractions (helix, turns, and sheets), for COMPASS data and sequences generated by both models. Most properties exhibit similar distributions across datasets. However, sequences generated by AmpGPT2 more closely match the COMPASS distributions than those generated by ProtGPT2. Notably, the molecular weights of sequences from COMPASS and AmpGPT2 fall between 2000 and 8000 Da, whereas ProtGPT2 sequences range from 7000 to 10,000 Da. Additionally, non-AMP sequences display higher variance in isoelectric point.

To explore the relationship between AMP and non-AMP sequences, we applied Uniform Manifold Approximation and Projection (UMAP)^23^ to reduce the dimensionality of the sequences based on their physicochemical properties. The COMPASS dataset served as a reference space, and projections of sequences generated by AmpGPT2 and ProtGPT2 were mapped accordingly.

**Fig. 3:**
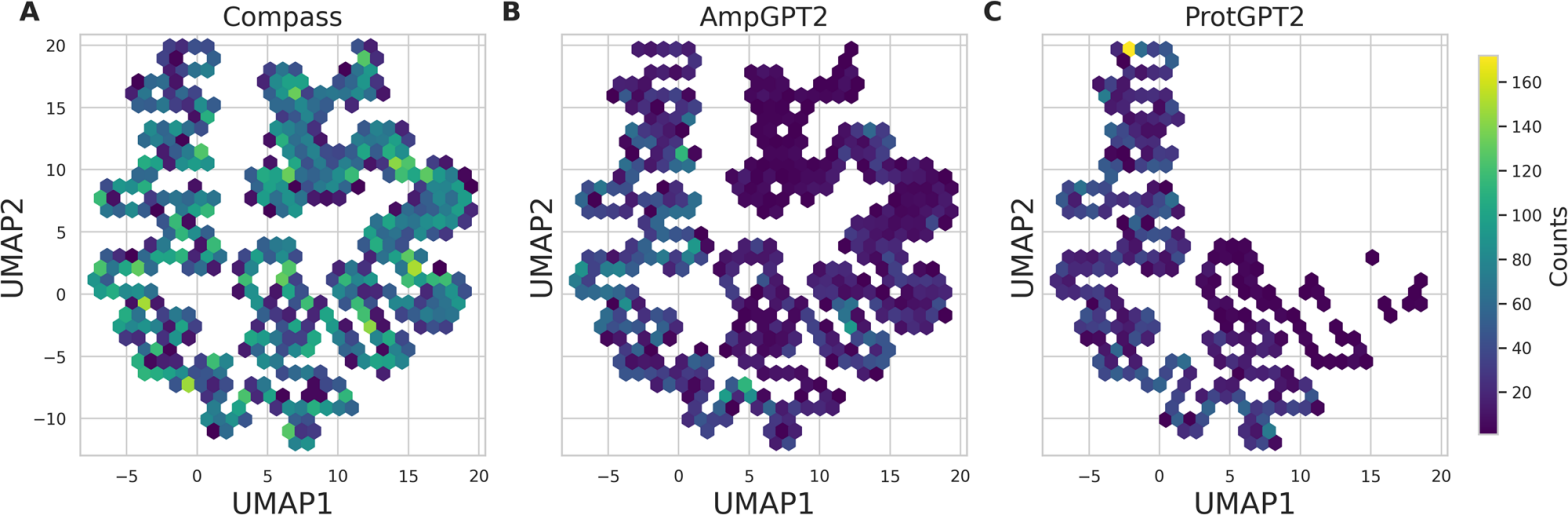
Physicochemical-property-based UMAP projections of peptide datasets. UMAP projections of **A** COMPASS antimicrobial peptides; **B** AmpGPT2-generated sequences; and **C** ProtGPT2-generated sequences based on calculated physicochemical properties. Each hexagonal bin represents peptide sequences located in the same region of the UMAP space, and the color intensity indicates the number of sequences within each bin.

As shown in Fig. 3, COMPASS sequences occupy the entire UMAP space. ProtGPT2-generated AMPs are concentrated primarily on the left side of the map, while AmpGPT2-generated AMPs extend across the map, covering the entire space, although with lower density than the COMPASS sequences.

### Experimental validation

**Fig. 4:**
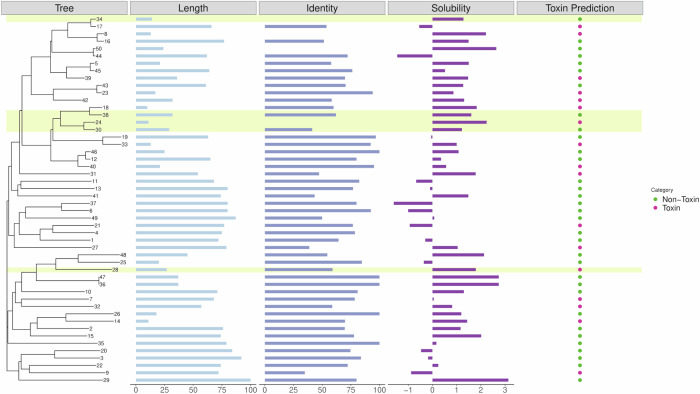
Phylogenetic tree generated with Clustal Omega of 50 randomly selected sequences from AmpGPT2. Each sequence is annotated with its length in amino acids, similarity to the top BLAST hit based on the COMPASS dataset, predicted solubility using CamSol (higher values indicate better solubility), and predicted toxicity using ToxinPred 3.0. Sequences highlighted in yellow were chosen for synthesis and experimental validation against *Klebsiella pneumoniae*.

Figure 4 shows a phylogenetic tree generated using Clustal Omega^24^ for 50 randomly selected sequences produced by AmpGPT2, annotated with their predicted solubility, similarity to COMPASS entries, and predicted toxicity as assessed by ToxinPred3^25^. The exact numerical values and predictions, including the ID for the best hit in COMPASS, are listed in the Supplement Table S7. Most sequences were predicted to be highly soluble, with the exception of sequences 6, 27, and 44, which exhibited poor solubility. Several sequences (8, 9, 24, 27, 30, 34, and 50) displayed low similarity to known AMPs or had no matches in COMPASS. All sequences had high predicted AMP scores ( > 0.9), well above the 0.5 threshold used to define potential AMP activity. From these, the five highlighted sequences (24, 28, 30, 34, and 38) were manually selected for synthesis and tested against *Klebsiella pneumoniae*, *Pseudomonas aeruginosa* and *Streptococcus pneumoniae*. Table 2 shows the predicted 3D structures of the selected AMP candidates. The color of the sequences shows the confidence of the prediction, with a darker color indicating more confidence. Among them, sequence 28 demonstrated strong antibacterial activity, while sequences 24, 30, 34, and 38 did not inhibit bacterial growth. AMP 24 was retained as a control alongside AMP 28 for comparative purposes.

**Table 2:** Selected sequences for synthesis and observed antimicrobial activity

ID	3D Prediction	Sequence	vs. *K. pneumoniae*	vs. *P. aeruginosa*
24		RRWKKRWWKWK	*p* < 0.01	*p* < 0.01
28		GFWGKLAKNAAKAAAKAALGALGQSGD	*p* < 0.001	*p* < 0.001
30		GIFSKIANVAKKVAGHLVPEVICKIAKTC	-	-
34		LKLLKWLWKWWKKD	-	-
38		GILSALKGAAKNVAMSLLDKLKCKIAGSCPGS	-	-

**Fig. 5:**
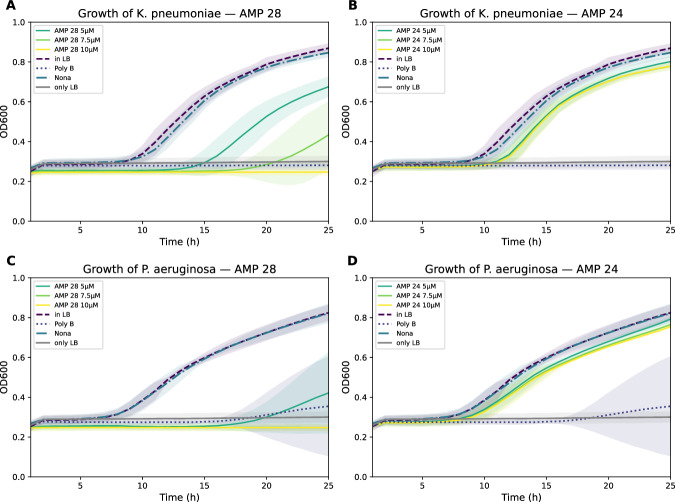
Growth kinetics of *Klebsiella pneumoniae* and *Pseudomonas aeruginosa* in the presence of antimicrobial peptides. Bacterial growth was monitored over 25 h by measuring optical density at 600 nm (OD600). Panels show growth of *Klebsiella pneumoniae* treated with AMP 28 (**A**) and AMP 24 (**B**), and *Pseudomonas aeruginosa* treated with AMP 28 (**C**) and AMP 24 (**D**). AMPs were tested at three concentrations (5, 7.5, and 10 μM). Control conditions included bacteria grown in LB medium without peptide (growth control, “in LB”), Polymyxin B as a positive antimicrobial control (“Poly B”), and a deactivated variant of Polymyxin B as a negative control (“Nona”). “Only LB" represents medium (Luria-Bertani broth) without bacteria to control for background signal. Lines represent mean growth curves across replicates, and shaded areas indicate variability between replicates. AMP 28 shows inhibitory activity against both bacterial species in a concentration-dependent manner, whereas AMP 24 does not substantially inhibit growth compared with the untreated control.

Figure 5 presents *Klebsiella pneumoniae* and *Pseudomonas aeruginosa* growth under varying AMP concentrations over a 25 h period. For both bacterial species, AMP 28 reduced bacterial growth relative to untreated controls, with a stronger inhibition observed at higher peptide concentrations. In contrast, AMP 24 produced only minor changes in growth compared to the bacteria-only condition. As expected, Polymyxin B strongly inhibited bacterial growth, whereas the inactive variant showed little to no inhibitory effect. The control, which only contained Luria-Bertani broth, remained stable throughout the experiment, confirming that increases in optical density in wells containing bacteria reflected bacterial growth rather than background signal.

Quantitative comparison of treatments using the area under the growth curve is summarized in Table 3. For *Klebsiella pneumoniae*, AMP 28 significantly reduced bacterial growth compared with untreated bacteria at all tested concentrations, with large effect sizes (Cohen’s *d* from − 9.18 to − 21.15). AMP 24 also produced statistically significant reductions in AUC, but with substantially smaller effect sizes. Similar results were observed for *P. aeruginosa*, where AMP 28 showed strong inhibitory effects across all tested concentrations, whereas AMP 24 produced comparatively modest reductions in bacterial growth. Polymyxin B showed strong antibacterial activity in both species, while the inactive Polymyxin derivative did not significantly differ from the bacteria-only control.

**Table 3:** AUC comparison of bacterial growth under peptide and control treatments

Treatment	Mean AUC diff (95% CI)	Cohen’s *d*	*p*-value
*Klebsiella pneumoniae*			
AMP 28 10 μM vs. bacteria	−6.87 (−7.47, −6.28)	−21.15	0.000005
AMP 28 7.5 μM vs. bacteria	−6.22 (−7.22, −5.21)	−11.33	0.000066
AMP 28 5 μM vs. bacteria	−4.71 (−5.52, −3.90)	−9.18	0.000203
AMP 24 10 μM vs. bacteria	−1.80 (−2.53, −1.07)	−6.54	0.002160
AMP 24 7.5 μM vs. bacteria	−1.56 (−2.27, −0.84)	−5.99	0.003296
AMP 24 5 μM vs. bacteria	−1.36 (−2.13, −0.59)	−4.86	0.006653
Polymyxin B vs. bacteria	−6.08 (−6.54, −5.62)	−27.16	0.000001
Non-active vs. bacteria	−0.28 (−0.77, 0.21)	−1.01	0.292879
AMP 28 10 μM vs. Polymyxin B	−0.79 (−1.24, −0.35)	−3.08	0.005654
AMP 28 7.5 μM vs. Polymyxin B	−0.14 (−1.14, 0.87)	−0.26	0.728163
AMP 28 5 μM vs. Polymyxin B	1.37 (0.56, 2.17)	2.76	0.012815
*Pseudomonas aeruginosa*			
AMP 28 10 μM vs. bacteria	−6.49 (−6.84, −6.14)	−36.92	0.000005
AMP 28 7.5 μM vs. bacteria	−6.46 (-6.81, −6.11)	−36.84	0.000005
AMP 28 5 μM vs. bacteria	−5.63 (−5.98, −5.28)	−30.87	0.000005
AMP 24 10 μM vs. bacteria	−1.88 (−2.47, −1.29)	−8.37	0.000757
AMP 24 7.5 μM vs. bacteria	−1.69 (−2.30, −1.08)	−7.41	0.001292
AMP 24 5 μM vs. bacteria	−1.44 (−2.04, −0.83)	−6.39	0.002383
Polymyxin B vs. bacteria	−5.46 (−5.82, −5.10)	−41.58	0.000001
Non-active vs. bacteria	−0.04 (−0.39, 0.31)	−0.30	0.933341
AMP 28 10 μM vs. Polymyxin B	−1.03 (−1.96, −0.10)	−2.21	0.042363
AMP 28 7.5 μM vs. Polymyxin B	−1.00 (−1.93, −0.07)	−2.15	0.043297
AMP 28 5 μM vs. Polymyxin B	−0.17 (−1.10, 0.76)	−0.36	0.879006

*Streptococcus pneumoniae* exhibited irregular growth kinetics across repeated assays, therefore limiting interpretability. Supplementary Fig. S8 shows an initial AMP concentration-dependent delay in *S. pneumoniae* growth, followed by a subsequent increase in bacterial growth that exceeded the untreated control.

Neither AMP 28 nor AMP 24 exhibited measurable hemolytic activity across the tested concentration range (0.1–100 μM)(Supplement Table S9). No additional cytotoxicity was observed when comparing the AMP-treated samples to the DMSO-treated samples (Supplement Table S10). Bactericidal activity of AMP 28 was observed against *Pseudomonas aeruginosa*. No colony formation was detected at 10 μM, whereas reduced colony numbers were observed at 7.5 μM, and extensive growth occurred at 5 μM (see Supplementary Fig. S11). While both *Klebsiella pneumoniae* and *Streptococcus pneumoniae* showed concentration-dependent inhibitory effects in the growth assays, reliable MIC and MBC values could not be determined in the corresponding assays under the tested conditions.

## Discussion

This study presents a computational framework for AMP discovery by integrating a curated database (COMPASS) with a fine-tuned generative language model (AmpGPT2). Our results highlight both the potential and current limitations of generative AI approaches for functional peptide design.

The development of effective AMPs through computational methods represents a critical frontier in addressing the global challenge of antimicrobial resistance. Here, we describe an undirected, foundational approach to generating AMP using transformer-based language models.

Fine-tuning the base ProtGPT2 model on curated AMP data yielded a fine-tuned variant, AmpGPT2, which substantially improved the generation of sequences with AMP-like computational properties. Specifically, 95.41% of sequences generated by AmpGPT2 were classified as AMPs by AMP Scanner, compared to 55.3% for the base model. Although CAMP predictions are generally lower, a clear improvement is also observed between the base and fine-tuned models, with CAMP yielding comparable AMP prediction rates for AmpGPT2-generated sequences and the training and validation data. The very high AMP Scanner rate for AmpGPT2 exceeds that of the training data (70% - 80%) and cannot be explained solely by data overlap, which remains limited to approximately 6% for AMP Scanner and 17% for CAMP. Instead, this reflects an intentional inductive bias introduced by AMP-specific fine-tuning, leading the model to preferentially generate sequences that strongly match learned AMP-defining features rather than memorizing training examples. This improvement was accompanied by biologically relevant sequence characteristics, including length distributions predominantly between 10 and 40 amino acids and physicochemical properties consistent with known AMPs. UMAP analysis further confirmed that AmpGPT2-generated sequences occupy a chemical space similar to natural AMPs, suggesting successful capture of relevant sequence-property relationships during fine-tuning. Entropy analysis highlights distinct generative behaviors between the models. ProtGPT2-generated sequences displayed higher token- and sequence-level entropy, consistent with greater uncertainty and broader exploration of protein sequence space. In contrast, AmpGPT2-generated sequences exhibited lower entropy, reflecting a more constrained and targeted generative distribution. Notably, entropy values were similar for AmpGPT2 training and validation sequences, indicating consistent model behavior across both seen and held-out data.

However, computational classification as an AMP does not guarantee biological activity. Experimental validation revealed that only one of the five computationally promising sequences demonstrated measurable antimicrobial activity under the tested conditions, specifically against *Klebsiella pneumoniae* and *Pseudomonas aeruginosa*. This discrepancy between computational prediction and biological function reflects broader challenges in de novo protein design and highlights the difficulty of predicting functional activity from sequence information alone.

AMP 28 exhibited clear, concentration-dependent antibacterial activity against both *Klebsiella pneumoniae* and *Pseudomonas aeruginosa*, producing marked reductions in bacterial growth across all tested concentrations and approaching the inhibitory effect observed for Polymyxin B at higher concentrations. In line with the plating observations, this effect translated into bactericidal activity against *Pseudomonas aeruginosa*, whereas for *Klebsiella pneumoniae,* the observed growth patterns primarily indicate inhibitory activity. In contrast, the OD measurement for AMP 24 indicated only weak growth inhibition, likely due to background absorbance of the compound and the solvent DMSO (Supplementary Table S5). For *Streptococcus pneumoniae*, we reproducibly observed a concentration-dependent delay in bacterial growth. However, after this initial lag phase, the bacteria appeared to recover and eventually reached optical densities comparable to untreated growth after prolonged incubation. This growth pattern indicates that *Streptococcus pneumoniae* is initially susceptible to the peptide but can recover after prolonged incubation. Such transient inhibition may reflect a primarily bacteriostatic mode of action under the tested conditions. However, concentrations above 10 μM were not further investigated due to the increased risk of unspecific secondary effects. Overall, although the tested AMP showed weaker effects against *Streptococcus pneumoniae* than against the tested Gram-negative bacteria, the observed growth inhibition suggests bacteriostatic potential in *Streptococcus*. We attribute the increased late-phase growth of AMP-treated streptococci to a lingering AMP-mediated effect that may positively select for bacteria with higher replication competence during the early phase of treatment. This may indicate a mode of action that is effective against both Gram-negative and Gram-positive bacteria.

Importantly, preliminary safety profiling indicated no detectable hemolytic activity and no additional cytotoxicity in HEK cells compared to the solvent control, suggesting that the observed antibacterial activity was not accompanied by overt membrane toxicity toward mammalian cells.

Notably, AMP 28 shares structural features with the dermaseptin family of AMPs (Alignment Supplement Figure S6), which are naturally occurring peptides from frog skin secretions known for broad-spectrum antimicrobial activity^26^.However, AMP 28 shows only approximately 70% sequence similarity to its closest dermaseptin family member. The natural diversity within the dermaseptin family, in which sequences can vary substantially while maintaining function, provides precedent for generating novel, functional variants through computational approaches. This supports the potential for generative models to explore previously uncharted AMP sequence space while preserving essential functional elements.

Our undirected approach generates sequences solely based on patterns present in natural AMP sequences, without explicit optimization for specific antimicrobial properties or targets, and relies exclusively on canonical amino acids without additional chemical modifications. While this limits specificity for particular applications, it offers several advantages: (i) broader exploration of sequence space beyond property-constrained regions, (ii) discovery of novel AMP families with unexpected characteristics, and (iii) establishment of a general foundation for subsequent refinement using directed methods. The generation of a functionally active peptide suggests that transformer-based models can capture meaningful biological patterns from sequence data alone. This foundational capability could serve as a starting point for directed optimization strategies, potentially combining broad exploratory capacity with targeted efficacy.

However, several limitations must be acknowledged. Experimental validation was restricted to five sequences and a limited bacterial panel, primarily focusing on Gram-negative pathogens, providing limited statistical power for general conclusions. The observed success rate (20%), while insufficient for immediate practical drug discovery, is nevertheless encouraging as a proof of concept. Large-scale random peptide screens report functional hit rates of approximately 1–2%^27^, indicating that our approach yields a substantial enrichment over chance-level discovery. Additionally, the COMPASS database may contain biases reflecting historical AMP discovery, potentially influencing the model’s learned patterns. Future work should incorporate more rigorously curated training data and expand validation to multiple bacterial strains, antifungal activity, and cytotoxicity assessment. Integration of undirected generation with directed optimization represents a promising avenue for future development. Parameter-efficient fine-tuning methods could enable rapid adaptation of the foundational model for specific targets or properties, potentially improving functional success while maintaining broad sequence exploration.

A direct benchmarking of AmpGPT2 against other generative AMP architectures, such as diffusion-based, variational autoencoder, or hybrid models, was not performed in this study. To the best of our knowledge, the field currently lacks a widely adopted, standardized, and architecture-agnostic benchmarking framework for AMP generation. This limitation is widely recognized in the AMP community, where fragmented datasets and inconsistent annotations have been shown to hinder computational approaches and slow the discovery of new candidates^28^. Reviews of deep generative models for peptide design describe a large diversity of model types (e.g., LSTM, VAE, GAN, diffusion) without a consensus on unified evaluation criteria, making controlled model comparison difficult^29^. Similarly, broader protein generative modeling research highlights the absence of unified evaluation benchmarks for foundation models, underscoring the general lack of standardized assessment frameworks^30^. Because existing studies rely on heterogeneous datasets, differing negative sampling strategies, and a variety of post hoc AMP classifiers, direct comparison of reported AMP prediction rates across studies is inherently limited. Establishing community-wide benchmarking datasets and evaluation protocols, therefore, represents an important direction for future work.

Despite these limitations, this work contributes to drug development research by demonstrating that generative AI can augment traditional antimicrobial discovery pipelines. The development of COMPASS as a community resource and demonstration that fine-tuned language models can generate peptides with genuine biological activity represents meaningful advances toward AI-assisted drug discovery. Given the urgent global need for new antibiotics, even modest success rates are potentially valuable when coupled with appropriate validation and development frameworks. Our undirected approach complements existing directed methods and may be particularly useful for exploring novel AMP families or serving as a starting point for subsequent optimization.

In conclusion, this study establishes the feasibility of undirected transformer-based models for functional AMP generation while highlighting the critical gap between computational prediction and biological validation. Future efforts should aim to bridge this gap by improving predictive methods, expanding experimental validation, and developing hybrid approaches that combine the strengths of both undirected and directed generation strategies.

## Methods

### COMPASS

For data collection, we developed scripts in Python 3.10 to retrieve information from the following databases: APD3^31^ (accessed February 2024), Bactibase^32^ (accessed September 2023), CAMP^33^ (accessed February 2024), DRAMP^34^ (accessed February 2024), dbAMP^35^, DBAASP^36^ (accessed February 2024), UniProt^37^ (accessed February 2024), LAMP2^38^, and YADAMP^39^ (accessed September 2023). We collected all sequences tagged as antimicrobial peptides from UniProt, including both reviewed (Swiss-Prot) and unreviewed (TrEMBL) sequences. The datasets were merged into a single JSON file using the sequence as a unique identifier.

The website was implemented using the JavaScript framework React (v. 17.0.2), along with react-dom (v. 17.0.2), react-scripts (v. 5.0.1), Grommet (v. 2.29.0), Material-UI (v. 4.12.4), and Material-Table (v. 1.58.2), following the design and implementation principles of CORDITE^40^ and LAMPPrimerBank^41^.

### Data preprocessing

A series of preprocessing steps was applied to the COMPASS dataset to retain only relevant sequences. First, duplicate entries were removed, and a set of valid amino acid characters was defined. Sequences containing non-standard characters (e.g., “B”, “J”, “U”, “O”, “X”, “Z”, whitespace) were excluded. To focus on AMP-relevant lengths, sequences shorter than 10 or longer than 100 amino acids were also removed. After removal of duplicate entries, exclusion of sequences containing non-standard amino acids, and restriction to sequences of 10–100 residues, approximately 30,000 high-confidence AMP sequences remained for model training. Finally, sequences were formatted in FASTA format, with each entry labeled as “<∣endoftext∣>” to create distinct training inputs. Newline characters were inserted every 60 amino acids, and the dataset was divided into training and validation sets in an 80/20 ratio.

### Development of AmpGPT2

To develop AmpGPT2, ProtGPT2 was fine-tuned on the curated AMP dataset from COMPASS. Fine-tuning enables the adaptation of a general model, trained on extensive protein data, to a more specific task with limited data, such as AMP generation. The fine-tuning was performed on the Marburger Computing Cluster (MaRC3a) using four NVIDIA A100 GPUs and the Hugging Face^42^run_clm.pyscript, designed for causal language model training. The Hugging Face Transformers library^42^, together with PyTorch^43^, provided the computational framework. Key parameters were set as follows: the base model and tokenizer nferruz/ProtGPT2^18^ were used, with a learning rate of 1 × 10^−5^, which, based on preliminary experiments, minimized overfitting while maintaining stable training and validation losses. All other hyperparameters were kept consistent with the original ProtGPT2 configuration. The batch size was adjusted to fit GPU memory constraints. Fine-tuning was conducted for 50 epochs, with evaluation performed at each epoch. The number of epochs was selected based on monitoring training and validation loss curves across different learning rate settings, which showed stable convergence without divergence between the two. Early stopping was not applied, as no increase in validation loss indicative of overfitting was observed during training. Training and validation loss curves were monitored for all model variants and are reported in the Supplementary Material (Fig. S4).

### Sequence generation and computational validation

Following fine-tuning, amino acid sequences were generated using AmpGPT2 to evaluate its effectiveness in producing AMP-like peptides. During sequence generation, a repetition penalty of 1.2 was applied, as this setting maximized the proportion of AMPs without substantially increasing sequence length. This penalty reduces repetitive patterns, promoting the generation of diverse, high-quality sequences. The do_sample parameter was set to True to enable multinomial sampling. A total of 10,000 sequences, ranging from 10 to 100 amino acids in length, were generated. The sequences were subsequently computationally checked using the Antimicrobial Peptide Scanner v2^44^ and CAMP^33^. These classifiers were applied strictly as post hoc computational filters and were not used during model training or sequence generation. The AMP Scanner combines convolutional and long short-term memory (LSTM) layers to classify sequences as AMPs or non-AMPs based on structural and compositional features, while CAMP uses several different models like Support Vector Machine (SVM), Random Forest (RF), Neural Net (NN) and Discriminant Analysis (DA) to classify sequences. For further evaluation, sequences generated by both ProtGPT2 and AmpGPT2 were compared using COMPASS, with several physicochemical properties calculated via the Biopython^45^ package. Sequence generation for ProtGPT2 was performed analogously to AmpGPT2. Model uncertainty was assessed by computing the average token-level and sequence-level entropy and negative log-likelihood (NLL) over validation, training, and generated sequences using each model’s predicted token probability distributions.

### Experimental validation

From the set of sequences generated by AmpGPT2, 50 sequences predicted to be AMPs by the AMP Scanner were randomly selected. These sequences were analyzed for similarity to existing entries in the COMPASS dataset using BLAST^46^. Solubility was predicted using CamSol^47^, toxicity was assessed with ToxinPred 3.0^25^, and three-dimensional structures were modeled using AlphaFold 3^48^. Based on these predictions, five sequences were selected for synthesis to experimentally evaluate their antimicrobial activity against *Klebsiella pneumoniae*, *Pseudomonas aeruginosa* and *Streptococcus pneumoniae*. Sequences were selected to maximize synthesis success while avoiding close similarity to existing AMP sequences. Shorter, more soluble candidates were favored, as longer sequences are more likely to fail synthesis due to strong secondary structures^49,50^.

*Klebsiella pneumoniae* was cultured following the protocol of Burt et al.^51^, *Pseudomonas aeruginosa* following Christe et al.^52^, and *Streptococcus pneumoniae* following Klabunde et al.^53^. To assess antimicrobial potency, bacteria were inoculated at an OD600 of 0.01 in the presence of AMPs at 5, 7.5, or 10 μM, with DMSO (Dimethyl sulfoxide) serving as the solvent control. Polymyxin B was included as a clinically established membrane-active antibiotic control against Gram-negative bacteria. Its nonactive variant, lacking antimicrobial activity, was used as an inactive control peptide. All assays were performed in parallel under identical experimental conditions. Bacterial growth was monitored over 25 hours by measuring OD600 on a Tecan M200 Pro spectrophotometer. All experiments were conducted in four biological replicates.

Hemolytic activity of the active AMP was assessed as described by Lindhauer et al.^54^. Erythrocytes were isolated from three healthy donors and incubated with the peptide at concentrations ranging from 0.1 μM to 100 μM for 1 h at 37 °C. A 1% Triton X-100 solution was used as a positive control. Hemolysis was quantified by measuring absorbance at 405 nm using a Tecan M200 Pro spectrophotometer and is reported as a percentage of total lysis relative to Triton X-100. All donors gave informed written consent on the use of their blood for scientific purposes (Marburg Ethics vote 110/19).

Cytotoxic effects of the active AMP on HEK (Human Embryonic Kidney) cells were assessed as previously described by Lindhauer et al.^54^. HEK cells were exposed to dilutions of the AMP ranging from 0.1 μM to 100 μM, with DMSO used as a solvent control. After 20 h of incubation, supernatants were collected, and extracellular lactate dehydrogenase (LDH) release was quantified using a Tecan M200 Pro spectrophotometer.

Growth curves were quantitatively compared using the area under the curve (AUC), computed per biological replicate over the selected time window. Differences in mean AUC were assessed using Welch’s two-sample *t*-test. Effect sizes were quantified using Cohen’s *d*, and 95% confidence intervals for the mean AUC differences were calculated using Welch’s method. To account for multiple comparisons, *p*-values were adjusted using the Benjamini-Hochberg false discovery rate correction. Statistical significance was defined as adjusted *p* < 0.05. MIC and MBC assays were performed following standard broth microdilution protocols. Bacteria were grown on appropriate agar plates (*Klebsiella pneumoniae*: MacConkey agar, *Pseudomonas aeruginosa*: LB agar, *Streptococcus pneumoniae*: sheep blood agar. Bacteria were grown overnight, and single colonies were picked for subculturing in Mueller-Hinton Broth overnight. Bacterial suspensions were then diluted 1: 1000 in Mueller-Hinton Broth and grown to a density of OD600=1. This suspension was added to 1:10 dilutions of the active AMP, ranging from 100 μM to 0.1 μM. Suspensions were grown for 1 h at 37 °C. Afterward, the lowest AMP concentration that inhibited visible growth (MIC) was selected, and the respective suspension was plated on LB agar, along with the last AMP concentration that still permitted bacterial growth. 24 h later, bacterial colonies were counted, and MBC was calculated.

## Supplementary information

Supplementary information

## Data Availability

The database can be downloaded at https://compass.imi.uni-muenster.de/data.json. A web interface is available under https://compass.imi.uni-muenster.de.

## Code availability

The AmpGPT2 model is available at https://imigitlab.uni-muenster.de/heiderlab/ampgpt2.
