# Supplementary material for: Harnessing generative AI for predicting and optimizing antimicrobial peptides against drug-resistant infections
Source: NPJ Antimicrob Resist. 2026 May 22;4:42. doi: 10.1038/s44259-026-00218-3 (PMC13230547; doi:10.1038/s44259-026-00218-3)
Supplement: Supplementary file 1 — Supplementary information [file 44259_2026_218_MOESM1_ESM.pdf]

Supplementary Material for Harnessing Generative AI for Predicting and Optimizing Antimicrobial Peptides Against Drug-Resistant Infections

Sandra Clemens, Hannah Franziska Löchel, Nico Häußer, Felix Wannemacher, Wilhelm Bertrams, Bernd Schmeck, Dominik Heider

October 2025

Abstract

This section contains a an analysis of the composition of the COMPASS database (Figure S1 - S3), the loss curves of AMPGPT2 (Figure S4, and the results of the analysis of the gerarated sequences by AMPGPT2 (Tabele S1, S2 and Figure S6)).

S1 Venn diagram of the databases of COMPASS

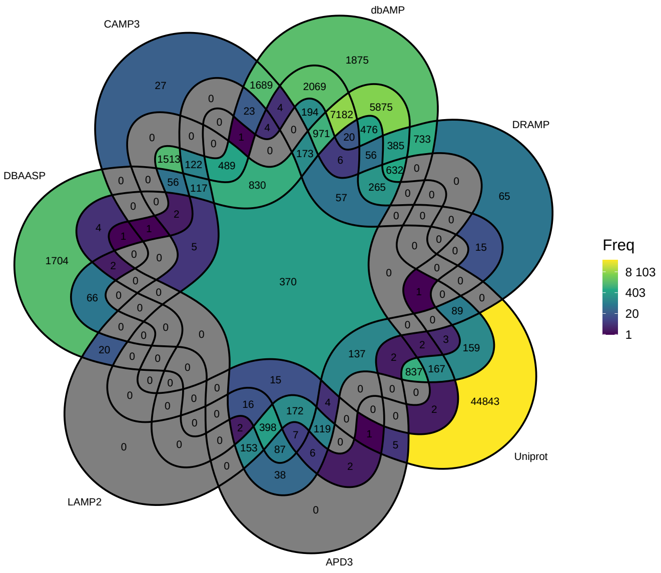

Figure S1: Overlap between the databases as a Venn diagram showing the number of unique and shared sequences. Not including the two databases with the least amount of sequences.

S2 Heatmap of the intersection of the databases of COMPASS

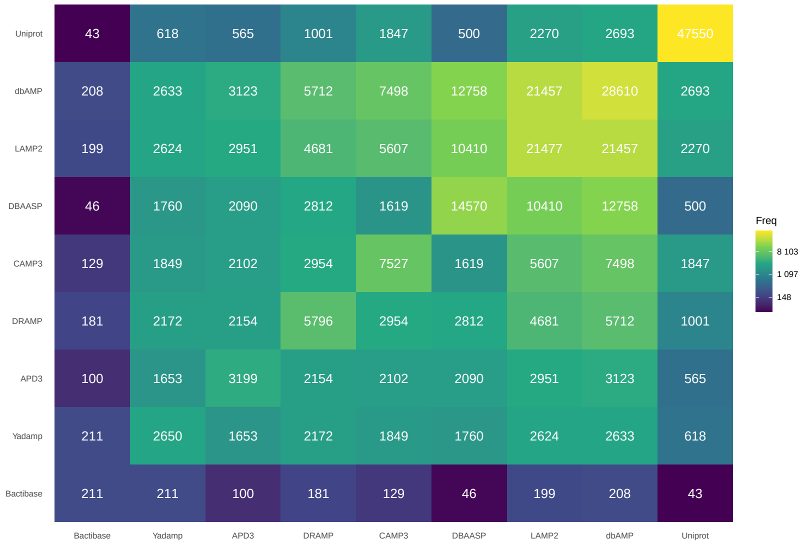

Figure S2: Shared and unique sequences across all databases used for COMPASS.

S3 UpSet plot Intersection of the databases of COMPASS

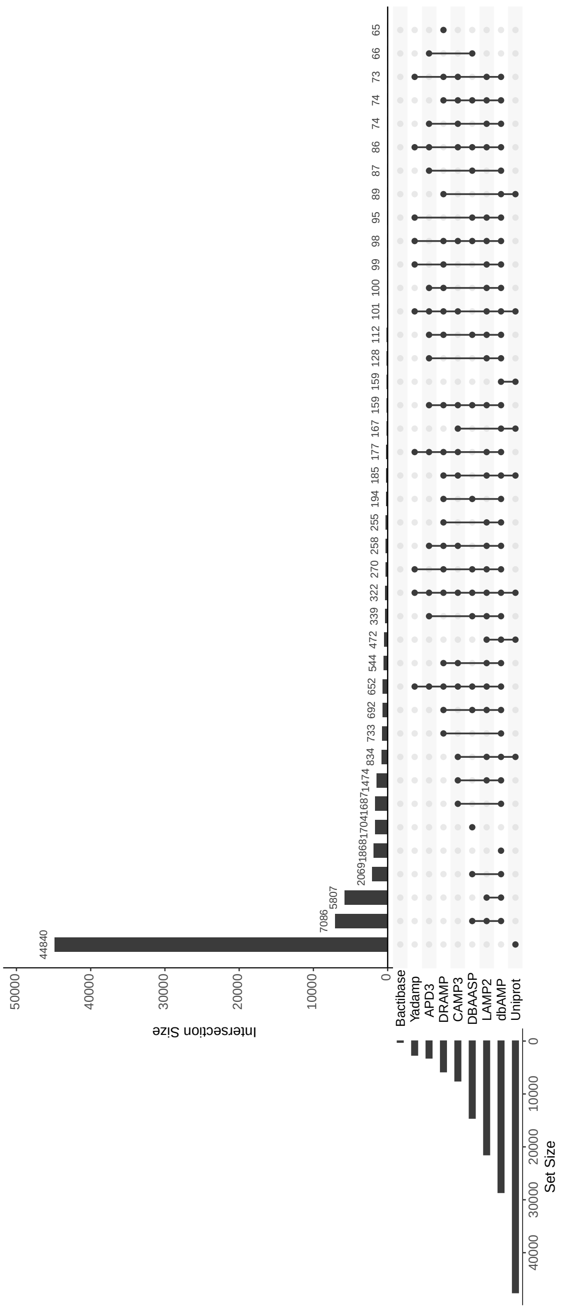

Figure S3: UpSet plot of the intersection among the databases included in COMPASS.

S4 Loss curves for the different learning rates

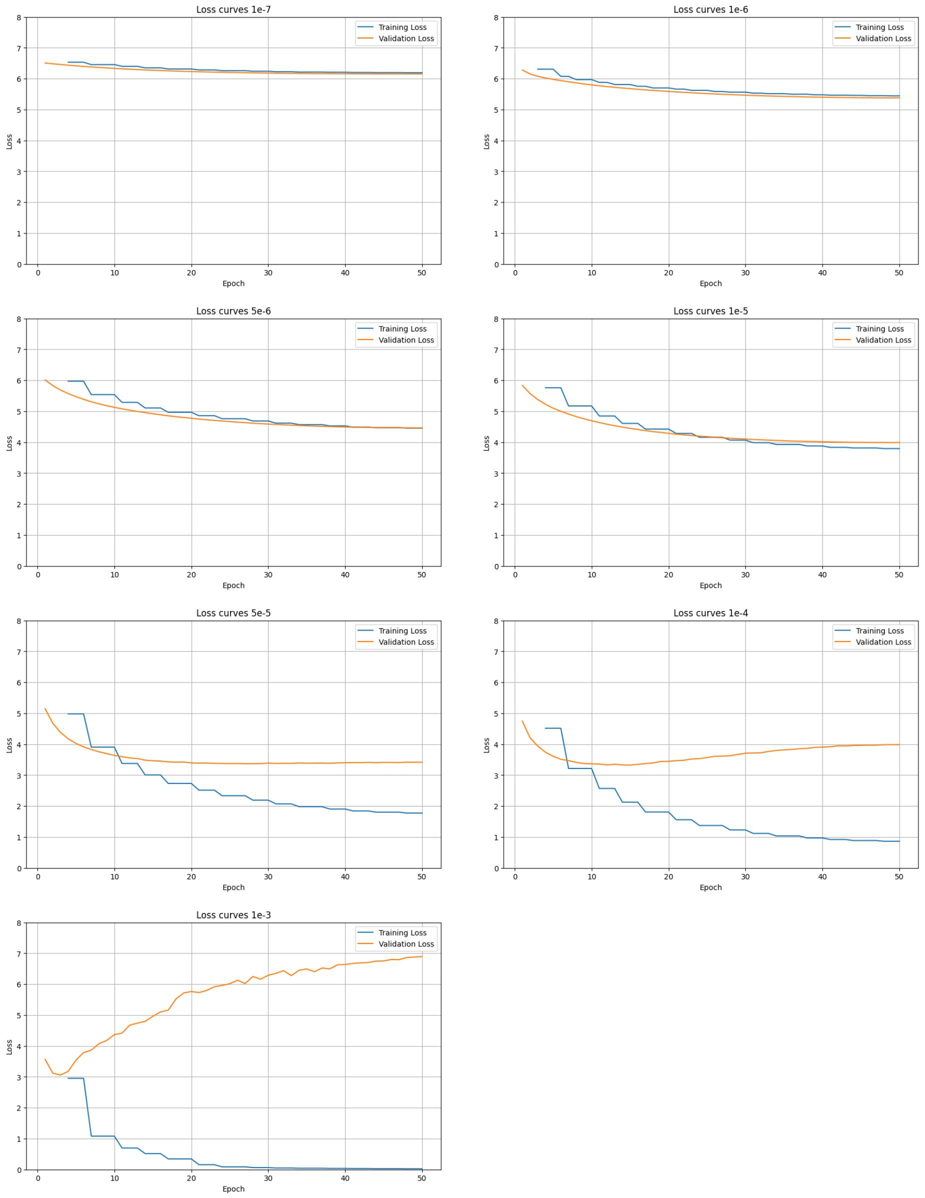

Figure S4: Loss curves for trainings and validation data over 50 epochs for different learning rates.

S5 Comparison growth of Klebsiella pneumoniae under different conditions

Table S1: Comparison of *Klebsiella pneumoniae* growth under different treatments

Comparison	Mean diff (95% CI) AUC	Cohen's d	p-value
AMP 28 10μM vs untreated	-6.35 (-6.83, -5.88)	-36.4	0.000156
AMP 28 10μM vs DMSO 10μM	-5.64 (-6.36, -4.93)	-23.7	0.000953
AMP 28 7.5μM vs untreated	-4.56 (-5.05, -4.08)	-21.6	6.56e-05
AMP 28 5μM vs untreated	-3.2 (-3.76, -2.64)	-13.1	0.0003
AMP 28 5μM vs DMSO 5μM	-2.67 (-3.22, -2.13)	-12.8	0.00118
AMP 24 10μM vs untreated	-1.73 (-2.23, -1.22)	-7.74	0.00122
AMP 24 10μM vs DMSO 10μM	-1.01 (-1.67, -0.356)	-3.68	0.0181
AMP 24 7.5μM vs untreated	-1.6 (-2.15, -1.04)	-6.56	0.00233
AMP 24 5μM vs untreated	-1.3 (-1.9, -0.704)	-5.05	0.006
AMP 24 5μM vs DMSO 5μM	-0.776 (-1.38, -0.173)	-3.47	0.0312
untreated vs DMSO 10μM	0.712 (0.0536, 1.37)	2.57	0.0422
untreated vs DMSO 5μM	0.526 (0.065, 0.987)	2.85	0.0388

S6 Alignment of AMP28 with some existing Dermaseptin-family Peptides

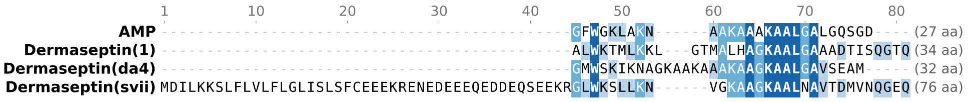

Figure S5: Alignment of AMP28 with some existing Dermaseptin-family Peptides.

S7 Sample Sequences generated with AMPGPT2

Table S2: Sample Sequences generated with AMPGPT2

Seq No	length	COMPASS ID	sequnce identity	solubility	Toxin Pred.
1	72	COM69980	64.29	-0.3	Non-Toxin
2	76	COM73454	69.74	1.16	Non-Toxin
3	92	COM05644	83.87	-0.18	Non-Toxin
4	75	COM68012	78.57	0	Non-Toxin
5	21	COM00733	57.9	1.5	Non-Toxin
6	80	COM70943	92.5	-1.01	Non-Toxin
7	68	COM54213	78.43	0.05	Toxin
8	13	-	0	2.21	Toxin
9	72	COM46646	34.92	-0.88	Toxin
10	71	COM14028	80.95	1.3	Non-Toxin
11	68	COM38867	82.35	-0.67	Non-Toxin
12	65	COM72849	80	0.36	Non-Toxin
13	80	COM62095	76.92	-0.1	Non-Toxin
14	11	COM02955	70	1.44	Toxin
15	74	COM73102	77.78	2.02	Non-Toxin
16	77	COM40089	51.52	1.5	Non-Toxin
17	66	COM41205	53.85	-0.55	Toxin
18	10	COM09159	60	1.83	Toxin
19	63	COM22850	96.83	-0.05	Non-Toxin
20	84	COM68606	74.71	-0.47	Non-Toxin
21	77	COM56601	76.81	-0.94	Toxin
22	74	COM55068	72.31	0.24	Non-Toxin
23	17	COM22675	94.12	0.87	Toxin
24	11	-	0	2.24	Toxin
25	20	COM25061	84.62	-0.36	Non-Toxin
26	18	COM17383	100	1.19	Non-Toxin
27	79	COM69829	38.82	1.04	Toxin
28	27	COM06312	59.09	1.79	Toxin
29	100	COM11337	80	3.14	Non-Toxin
30	29	COM05296	41.38	1.22	Non-Toxin
31	54	COM47776	47.37	1.79	Toxin
32	57	COM38005	58.82	0.82	Toxin
33	13	COM10279	92.31	1	Toxin
34	14	-	0	1.28	Non-Toxin
35	79	COM48014	100	0.17	Non-Toxin
36	37	COM08232	100	2.74	Non-Toxin
37	80	COM57554	80	-1.6	Non-Toxin
38	32	COM01796	62.07	1.61	Non-Toxin
39	36	COM66057	70	1.48	Toxin
40	21	COM72851	95.24	0.57	Toxin
41	74	COM08694	43.48	1.48	Non-Toxin
42	32	COM73733	58.33	1.31	Toxin
43	61	COM57673	70.49	1.27	Non-Toxin
44	62	COM41852	72.22	-1.46	Non-Toxin
45	64	COM22164	76.36	0.52	Non-Toxin
46	25	COM72887	100	1.08	Non-Toxin
47	37	COM08232	100	2.74	Non-Toxin
48	45	COM32128	54.55	2.13	Non-Toxin
49	87	COM56595	50	0.08	Non-Toxin
50	24	-	0	2.64	Non-Toxin

S8 Growth of *Streptococcus pneumoniae* treated with AMP28 and 24

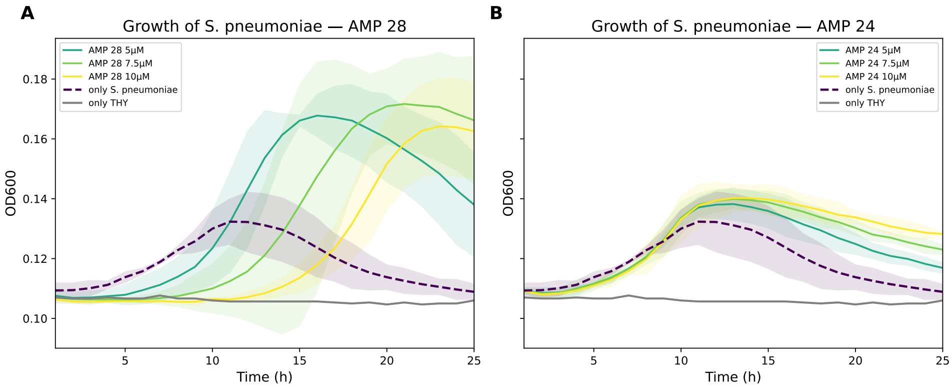

Figure S6: Growth of *Streptococcus pneumoniae* treated with AMP28 and 24.

S9 Hemolysis Assay

Table S3: Hemolytic activity of AMP28 and AMP24 against human erythrocytes. Hemoysis is expressed as percentage relative to complete erythrocyte lysis induced by Triton X-100 (100% control).

Concentration (μM)	AMP 28 (% hemolysis)	AMP 24 (% hemolysis)
100	0.013	0.010
10	0.008	0.008
1	0.007	0.008
0.1	0.006	0.007

S10 LDH Cytotoxicity Assay

Table S4: Cytotoxicity measured by LDH release assay. Values represent percent cytotoxicity relative to maximum LDH release control.

Condition	AMP28 (%)	AMP24 (%)
100μM	54.3	57.7
10μM	44.5	37.5
1μM	38.0	49.1
0.1μM	54.4	45.2
Untreated control	43.9	
DSMO (1 μl)	43.2	
DSMO (5 μl)	48.2	

S11 Determination of Minimum Bactericidal Concentration (MBC) against *Pseudomonas aeruginosa*

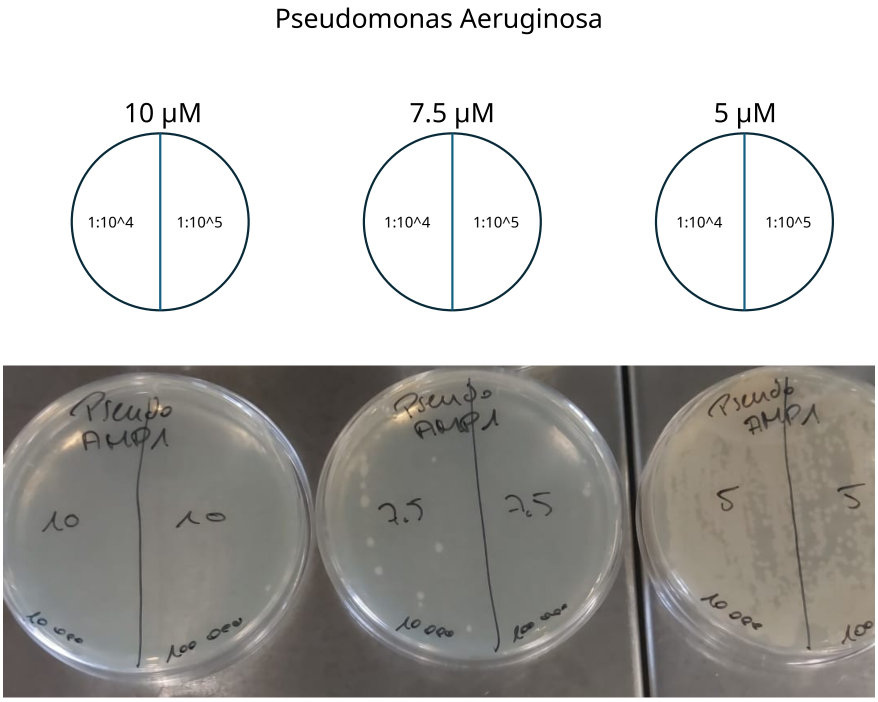

Figure S7: Agar plates used for determination of the minimum bactericidal concentration (MBC) of AMP28 against *Pseudomonas aeruginosa*. Cultures treated with AMP 28 at 10 μM, 7.5 μM, and 5 μM were plated at diluations of 1:10^4 and 1:10^5 each. No colony growth was observed at 10 μM, while reduced colony numbers were visible at 7.5 μM and extensive growth occured at 5 μM. These results indicate bactericidal activity of AMP 28 against *Pseudomonas aeruginosa* at concentrations $\geq$10 μM.
